# Imatinib Use in Pregnancy

**DOI:** 10.5505/tjh.2012.82542

**Published:** 2012-12-05

**Authors:** Michael J Webb, Debbie Jafta

**Affiliations:** 1 Division of Clinical Haematology, Department of Internal Medicine, Faculty of Health Sciences, University of the Free State, Bloemfontein, South Africa; 2 Department of Haematology and Cell Biology, National Health Laboratory Service, Faculty of Health Sciences, University of the Free State, Bloemfontein, South Africa

**Keywords:** Imatinib, Tyrosine kinase inhibitors, Chronic myeloid leukemia, Pregnancy

## Abstract

The outcome in patients with chronic myeloid leukemia (CML) has dramatically improved over the last decade due to the widespread use of novel tyrosine kinase inhibitors such as imatinib. As overall survival has improved, the number of women with CML that wish to become pregnant has increased. As such, attending physicians are faced with a dilemma - continue life-prolonging medication to treat the cancer, or interrupt its use due to its potential teratogenicity. Herein we describe 2 CML patients that gave birth. Case 1 was managed via substitution of imatinib with interferon. The patient’s child underwent genetic evaluation at age 3 years, achieved normal developmental milestones, and despite being shorter than his peers was proportional. In terms of morphology, the child had clinodactyly, short fifth fingers, and slightly downward slanting palpebral fissures, but otherwise appeared normal. In case 2 imatinib was continued throughout the pregnancy. This patient’s child underwent postpartum evaluation by a geneticist and was observed to be morphologically normal, except for clinodactyly and low-set ears.

**Conflict of interest:**None declared.

## Abstract (tr)

Kronik myeloid lösemili (KML) hastaların sonuçları son on yıl içinde yeni tirozin kinaz inhibitörlerinin yaygın olarak kullanılmasıyla dramatik bir düzelme göstermiştir. Daha iyi genel sağkalımla hamile kalan KML hastası kadınların sayısında bir artış görülmüştür. İlgili doktor bir ikilemle karşı karşıyadır: kanseri tedavi etmek için yaşamı uzatıcı ilaçları devam ettirme veya olası teratojenisite nedeniyle kullanımlarını kesme. KML’li kadınlarda iki başarılı hamileliği sunuyoruz . Birinci vaka imatinib yerine interferon kullanılmasıyla takip edildi. Bu vakada çocuğa üç yaşındayken genetik değerlendirme yapıldı. Normal gelişimsel referans noktalarına ulaşmıştı ama yaşıtlarından fiziksel olarak daha küçüktü. Ancak orantılar normaldi. Morfolojik olarak klinodaktili, kısa 5. parmaklar ve palpebral fissürlerde hafif aşağıya doğru eğim olması dışında normal görünüyordu. İkinci vakada imatinib hamilelik boyunca devam ettirildi. Çocuğa postpartum bir genetik uzmanı tarafından değerlendirme yapıldı ve klinodaktili ve alçak yerleşimli tırnaklar dışında morfolojik olarak normal bulundu.

## INTRODUCTION

Chronic myeloid leukemia (CML) is a myeloproliferative neoplasm associated with the Philadelphia chromosome. The Philadelphia chromosome results from the reciprocal translocation of chromosomes 9 and 22, leading to production of the BCR-ABL fusion protein. This fusion protein is thought to be the initiating event in the pathogenesis of CML. Inhibition of this protein was a logical therapeutic target and led to the development of imatinib mesylate (Gleevec^®^, Novartis, Basel, Switzerland), a firstgeneration tyrosine kinase inhibitor. 

The International Randomized Study of Interferon Versus STI571 (IRIS) showed conclusively that imatinib 400 mg was superior to the standard care offered at the time of the study [1]. To date, imatinib remains the optimal firstline therapy for patients with CML. In terms of monitoring patients treated with imatinib, time-related improvement in hematological, cytogenetic, and molecular markers of disease is important [2]. To achieve optimal outcome, imatinib should be administered at 400 mg po /day [3]. Treatment compliance is important and dose interruptions have a negative impact on outcome [4]. 

With the advent of imatinib, CML has been transformed from a universally fatal disease to a disease with an estimated 7-year overall survival of 86% [5]. Along with the increase in the number of CML patients experiencing long-term survival come additional challenges, such as an increase in the number of female patients that want to become pregnant. This poses a potential predicament for attending physicians, as based on limited data available in human and animal models, it is recommended that imatinib (a life-saving therapy) not be administered to pregnant women [6]. Herein we describe 2 female CML patients treated with imatinib therapy that subsequently gave birth.

## CASE 1

A newly married 26-year-old female presented to her general physician with headache, depression, and splenomegaly. She was subsequently diagnosed with Philadelphia chromosome-positive chronic-phase CML in November 2004. Her full blood count findings were as follows: WBC count: 300 x 10^9^/L (normal range: 4.0-11.0 x 10^9^/L); Hb: 10.0 g/dL (normal range: 11.5-16.0 g/dL), Plt count: 249 x 10^9^/L (normal range: 150-450 x 10^9^/L). The differential count included a neutrophil count of 90 x 10^9^/L (normal range: 2.0-7.5 x 10^9^/L) and an increase in immature granulocytes. Her peripheral blast count was 3% (normal value: 0%). The patient’s bone marrow was hypercellular without an increase in the blast count. 

Clinical examination showed that her spleen was enlarged to 6 cm below the costal margin. She was initially started on hydroxyurea 2 g/d p.o. until imatinib could be procured via the Glivec^®^ International Patient Assistance Program (GIPAP). She was started on imatinib 400 mg/d in June 2005, and achieved complete hematological response in September 2005 and complete cytogenetic remission (CCyR) in June 2006. In February 2007 she wanted to become pregnant. Qualitative PCR was performed in March 2007, which was negative for the BCRABL gene rearrangement. Following patient consultation, imatinib treatment was withdrawn in April 2007 and subcutaneous (SQ) interferon-α was initiated at a dose of 3 MU/m^2^ 5 times/week. 

Contraception was then stopped and 2 months later she was pregnant. The patient remained on interferon-α through out her pregnancy, without the need for dose reduction due to side effects. The pregnancy was carefully monitored in conjunction with the patient’s obstetricians and she delivered a 1540-g boy in November 2007 via a Caesarian section at 33 weeks of gestation. The premature birth was due to pre-eclampsia. As a neonate the child was diagnosed with a patent foramen ovale and patent ductus arteriosus, which were considered complications of the premature delivery. The child then developed grade 3 hyaline membrane disease and hospital-acquired pneumonia. The congenital heart lesions resolved without intervention and the child fully recovered from his pulmonary complications. 

The patient remained in CCyR for the duration of her pregnancy. Imatinib was reinstituted post delivery and the patient was advised not to breastfeed. The patient maintained CCyR and exhibited a continuous decrease in her BCR-ABL transcript level, although she still did not achieve a major molecular response (not adjusted to the international scale). 

The patient’s child was evaluated by a geneticist at age 3 years. The boy achieved normal developmental milestones, but was just below the third percentile for height and weight—he was, however, proportional. In terms of morphology, the child had clinodactyly, short fifth fingers, and slightly downward slanting palpebral fissures, but otherwise appeared normal.

## CASE 2

A 15-year-old female was diagnosed with Philadelphiachromosome-positive chronic-phase CML in 2004. Hersymptoms included fatigue, peripheral edema, and bilateralhip pain. She presented with a WBC count of 347 x10^9^/L, Hb of 6.0 g/dL, and a Plt count of 16 x 10^9^/L. Her differential count included 27% neutrophils, 11% metamyelocytes, 45% myelocytes, and 3% blasts. Her bonemarrow was hypercellular without an increase in the blastcount. Clinical examination showed that her spleen wasenlarged to 4 cm below the costal margin. She was initiallystarted on hydroxyurea 1.5 g/d p.o. until imatinibcould be procured via the GIPAP program. She achievedcomplete hematological remission after 3 months of treatmentand cytogenetic remission after 12 months. After 18months of treatment she had not achieved major molecularresponse. The imatinib dose was not increased becausethe patient developed neutropenia, which required a doseinterruption.

In October 2009 (at age 21 years), having subsequentlyattained major molecular response, the patient discoveredthat she was 8 weeks pregnant. The treatment optionswere discussed with the patient and she decided to continuetaking imatinib 400 mg/d during the pregnancy. Thepregnancy was carefully monitored in conjunction withher obstetricians and she delivered a boy in March 2010via Caesarian section, which was indicated due to intrauterinegrowth restriction. At birth the infant weighed1980 g and had APGAR scores of 9 and 10. The child wasevaluated by a geneticist and except for clinodactyly andlow-set ears the child was morphologically normal. Themother was advised against breastfeeding.

## DISCUSSION

Treating patients with CML that wish to become pregnant is challenging for a number of reasons. There is no therapy that can be offered to a pregnant woman that is both completely safe and effective, and clinicians are therefore faced with the challenge of balancing the safety of the mother and treating her malignancy against the safety of the unborn child. Data on pregnancy outcomes in patients treated with imatinib are limited. One of the largest collections of data on the effect of imatinib on pregnancy outcome was compiled by Pye et al. [7]. They obtained data on 180 pregnancies in patients receiving imatinib from attending doctors that reported to the Novartis Pharmaceutical Company in Switzerland, the Hammersmith Hospital in London, or the M. D. Anderson Cancer Center in Huston. Fetal abnormalities were observed in 12 cases. A matter of great concern highlighted by the researchers was the increased preponderance of bony abnormalities, which indicated a potential correlation with imatinib treatment. Both of the presented patients’ children had clinodactyly, which can be considered a normal variant or a minor malformation, as its incidence varies from 1% to 19% [8]. Clinodactyly was not considered to be causally linked to the use of imatinib in the presented cases. 

Data from the Stop Imatinib (STIM) Trial indicate that imatinib can be discontinued in a select subgroup of patients that achieve complete molecular response and maintain it for 2 years [9]. This may well be the ideal method to manage a pregnancy, but may not be applicable to all patients. Patients with disease that is not optimally controlled, as per the European LeukemiaNet (ELN) guidelines [2], may be even more difficult to manage. In such cases continuation of therapy with close monitoring of the pregnancy may facilitate continuation of effective therapy, but pose the risk of adverse fetal outcome. 

Other modalities of therapy that have been used include hydroxyurea, interferon-α, and leukapheresis [10,11,12], each of which is potentially problematic during pregnancy, although interferon may be a safer option though less efficacious than imatinib [13,14]. We described 2 CML patients that gave birth following different approaches to the management of their pregnancies. Case 1 was managed in what may be considered a more classical approach-interruption of imatinib and the use of interferon. This option resulted in a successful pregnancy without the loss of disease control. Case 2 was more complex because the patient failed to achieve an optimal response, according to published guidelines [2]. Consequently, there was concern about disease progression and resistance should therapy be interrupted. In the light of normal fetal ultrasonographic findings at the end of the first trimester, and consultation with colleagues and the patient, imatinib was continued during the pregnancy with close monitoring, which resulted in a favorable outcome. 

According to Pye et al. [7] and preclinical animal models [6], it is important that women be made aware of the potential complications of therapy for CML during pregnancy; however, in selected cases continuation of imatinib with close monitoring of the pregnancy may facilitate control of the disease and delivery of a normal infant. 

**Acknowledgement**

The authors thank Dr. Daleen Struwig, University of the Free State, Faculty of Health Sciences, for technical and editorial preparation of the manuscript for publication. 

**Conflict of Interest Statement**

None of the authors have any conflicts of interest, including specific financial interests, relationships, and/or affiliations, relevant to the subject matter or materials included.
